# Statistical Analysis for Collision-free Boson Sampling

**DOI:** 10.1038/s41598-017-15596-y

**Published:** 2017-11-10

**Authors:** He-Liang Huang, Han-Sen Zhong, Tan Li, Feng-Guang Li, Xiang-Qun Fu, Shuo Zhang, Xiang Wang, Wan-Su Bao

**Affiliations:** 1 Henan Key Laboratory of Quantum Information and Cryptography, Zhengzhou Information Science and Technology Institute, Henan Zhengzhou, 450000 China; 2 CAS Centre for Excellence and Synergetic Innovation Centre in Quantum Information and Quantum Physics, University of Science and Technology of China, Hefei, Anhui 230026 China; 3 Hefei National Laboratory for Physical Sciences at Microscale and Department of Modern Physics, University of Science and Technology of China, Hefei, Anhui 230026 China

## Abstract

Boson sampling is strongly believed to be intractable for classical computers but solvable with photons in linear optics, which raises widespread concern as a rapid way to demonstrate the quantum supremacy. However, due to its solution is mathematically unverifiable, how to certify the experimental results becomes a major difficulty in the boson sampling experiment. Here, we develop a statistical analysis scheme to experimentally certify the collision-free boson sampling. Numerical simulations are performed to show the feasibility and practicability of our scheme, and the effects of realistic experimental conditions are also considered, demonstrating that our proposed scheme is experimentally friendly. Moreover, our broad approach is expected to be generally applied to investigate multi-particle coherent dynamics beyond the boson sampling.

## Introduction

Quantum computers offer the promise of efficiently solving certain problems, such as factorization^1^, that are intractable for classical computers. To date, much significant progress for various quantum systems has been made towards scalable quantum computing^2^, such as trapped ions^3,4^, atoms system^5,6^, linear optics^7–11^, nuclear magnetic resonance^12^, and superconducting system^13–15^. We are confident that in the future, the dream of large-scale quantum computer will eventually come ture. Recently, considerable interest has been generated concerning the construction of non-universal quantum computers, which demand less physical resources but can experimentally demonstrate the quantum supremacy over classical computers in the near term^16^, which will be a milestone for large-scale quantum computer.

In a major breakthrough Aaronson and Arkhipov proposed a problem called boson sampling^17^, which is intractable for classical computers but can be naturally and efficiently solved on a specialized photonic quantum simulator. Unlike full linear optics quantum computing (LOQC)^18^, the boson sampling device requires only single-photon sources, passive linear optics, photodetection, and especially much less resources. Thus, boson sampling is considered as a leading candidate to experimentally demonstrate the quantum supremacy of quantum machines in the near future^19^. So far, a number of elegant boson sampling experiments has been achieved with linear optics on a small scale^20–30^. With the rapid progress of experimental technology, we are optimistic that quantum supremacy is no longer far from us.

The core hardness of classically simulating boson sampling lies at calculating the permanent of an arbitrary complex matrix, which is a #*p*-complete computational problem^31^. Quantum simulation of boson sampling has a great advantage that needs not to calculate the permanent. However, after obtaining samples, another major problem arises: how to efficiently certify the correctness of the experimental results? A number of verification methods have been developed^24,25,32–36^, but the application scenarios of these methods are limited. For example, the row-norm estimator^32^ is efficiently computable, but can be only used to distinguish between boson sampling and uniform sampling. Bayesian probabilistic analysis^33^ or likelihood ratio test^24^ promises to exclude any types of sampling, but requires calculating classically intractable matrix permanents. Recently, a novel statistical approach towards the certification of boson sampling experiments has been proposed by Walschaers *et al*.^37^, which provides an efficient and reliable strategy for distinguishing different particle types, such as bosons, distinguishable particles, fermions, or simulated bosons, by analyzing the two mode correlation function of the output state. In their approach, all types of the output need to be analyzed, including collision-free events (one photon per output-mode) and collision events (multiple photons per output-mode). However, measuring collision events must require photon-number-resolving detectors or the combination of beam splitters and multiple detectors, which increases the difficulty for practical experiments, especially for large-scale boson sampling. This shortcoming limits the practicability of the method.

To overcome this obstacle, here we extend the statistical analysis approach to the collision-free boson sampling. Since photon-number-resolving detectors are not necessarily required for the collision-free boson sampling, our scheme is more experimentally friendly than the previous scheme. Furthermore, compared to the previous scheme, more types of sampling are simulated and distinguished, which shows the universality of our scheme. Finally, to show the practicability and reliability of our scheme, numerical simulations are performed to demonstrate our scheme could still work with limited samples, and our scheme could tolerate a moderate amount of experimental noise while strong noise will be identified and excluded. All of these excellent features will provide us an effective and practical way for certifying collision-free boson sampling experimentally.

## Theory

Before introducing our proposed scheme, we first briefly describe the boson sampling problem and give some basic definitions. In the typical boson sampling model, we often consider an *m*-mode linear quantum network *U* with *n* single photons in as the input at distinct modes *k*_1_, …, *k*_*n*_. These *n* photons are evolved via the linear quantum network *U*, and finally be sampled via coincidence photodetection as the output state at modes *j*_1_,…,*j*_*n*_. In fact, the output can be divided into two types, one called collision-free event if ${j}_{1}\ne {j}_{2}\ne {j}_{3}\ne \cdots \ne {j}_{n}$, and the another one called collision event if *j*_*a*_ = *j*_*b*_ for some *a*, *b* ∈ [1, …, *n*], *a* ≠ *b*. In this work, we will consider only the collision-free boson sampling, which is defined as the boson sampling with post-selecting only the collision-free events.

**Figure 1:**
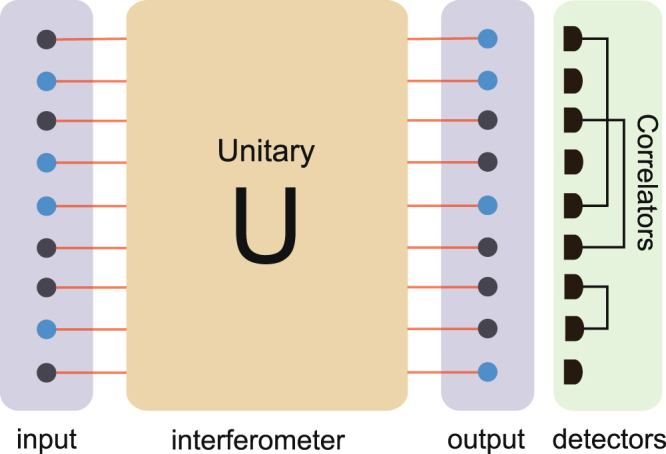
The boson sampling model and framework of two mode correlation function measurement. *n* single photons (colored by blue and *n* = 4 here) are injected into a *m* optical modes network *U*. The output are sampled via coincidence photodetection. To certify the results, we measure the two mode correlation in the experiment. The blocks with different colors represent the input, interference, output and measurement stages.

In general, to certify the collision-free boson sampling, usually we need to construct a suitable discriminator. In fact, the sampling problem is directly related to the probability of output, so we can employ the methods and parameters in probability statistics to construct the discriminator. In the probability theory, correlation functions play a central role, and the full knowledge of correlation functions implies full knowledge of probability distribution. Thus, we can use the correlation functions to analyze the collision-free boson sampling. However, it is time-consuming to compute all the correlation functions. Here, we consider only the two mode correlation function

1 $${C}_{ij}=\langle {\hat{n}}_{i}{\hat{n}}_{j}\rangle -\langle {\hat{n}}_{i}\rangle \langle {\hat{n}}_{j}\rangle $$

of output modes *i* and *j* ^38^, where ${\hat{n}}_{i}={a}_{i}^{\dagger }{a}_{i}$ is the particle number operator. It has been proved that two mode correlation functions can be efficiently computed^37^. In the experiments, the details of the two mode correlation function measurement procedure are shown in Fig. 1. Below, we show that we can extract useful and robust information from the two mode correlation functions, and use them to distinguish the sampling results of genuine collision-free boson sampling and other types of sampling.

Suppose we consider sufficiently many choices for output modes *i*, *j* ∈ {1, …, *m*}, *i* < *j*, and compute the corresponding two mode correlation function *C*_*ij*_. Then a set of data *C* = {*C*_*ij*_|*i*, *j* ∈ {1, …, *m*} and *i* < *j*} could be obtained, on which we can do some further statistical analysis to construct a proper discriminator, which could clearly reveal the different characteristics of collision-free boson sampling and other types of sampling and then could be used to certify the genuine collision-free boson sampling. In our proposed scheme, two statistical parameters, coefficient of variation (*CV*) and the skewness (*S*)^39^

2 $$CV=\frac{\sqrt{{\rm{E}}({C}^{2})-{\rm{E}}{(C)}^{2}}}{{\rm{E}}(C)},S=\frac{{\rm{E}}({C}^{3})-3{\rm{E}}(C){\rm{E}}({C}^{2})+2{\rm{E}}{(C)}^{3}}{{({\rm{E}}({C}^{2})-{\rm{E}}{(C)}^{2})}^{\mathrm{3/2}}}$$

are considered, where E(*C*) denotes the function of averaging over the set of data *C*. In statistics. the *CV* is a standardized measure of dispersion of a probability distribution or frequency distribution, and the *S* is a measure of the asymmetry of the probability distribution of a real-valued random variable about its mean. In order to find some useful regularities, we simulate the collision-free boson sampling and other types of sampling (distinguishable particles sampling, fermions sampling, uniform sampling, mean-field sampling^34^, and thermal state sampling^40^) for 200 different *U* matrices (we generate and choose the *U* matrices using method proposed by Francesco^41^ and Maris^42^). In our simulations, assume the input mode is *I* = {*i*_1_, *i*_2_, ..., *i*_*n*_}, the collision-free output mode is *O* = {*o*_1_, *o*_2_, ..., *o*_*n*_}, the probabilities of the output mode *O* for boson sampling ${P}_{I,O}^{B}$, distinguishable particles sampling ${P}_{I,O}^{D}$, fermions sampling ${P}_{I,O}^{F}$, and uniform sampling ${P}_{I,O}^{U}$ are

3 $${P}_{I,O}^{B}=|Per({U}_{I,O}{)|}^{2}$$

4 $${P}_{I,O}^{D}=Per(|{U}_{I,O}{|}^{2})$$

5 $${P}_{I,O}^{F}=|Det({U}_{I,O}{)|}^{2}$$

6 $${P}_{I,O}^{U}=\frac{1}{(\begin{array}{c}m\\ n\end{array})}$$

where *U*_*I*,*O*_ is an *n* × *n* sub-matrix of *U* related to the input *I* and output *O*, *Per* and *Det* are the permanent and determinant of a matrix, respectively. The probabilities of the output mode *O* for mean-field sampling and thermal state sampling could be calculated according to Tichy *et al*.^34^ and Rahimi-Keshari *et al*.^40^. According to the probability, we then could simulate the each type of sampling using the Monte Carlo method.

**Figure 2:**
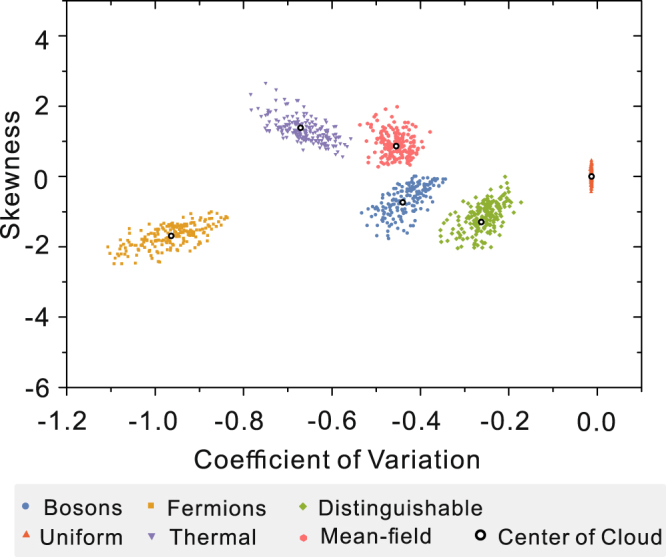
Numerical simulation results of the statistical analysis scheme. In the simulations, *m* = 20, *n* = 8. We simulated 200 different *U* matrices for each type of sampling. Different color points represent the statistical results of different types of sampling, and the black circles represent the mean value of each point cloud generated by each type of sampling. It is clear to see that the same sampling will be gathered into a cloud, and points for different types of sampling are all separated from each other, showing clearly that different samplings can be distinguished.

For all simulations, we let *m* = 20, *n* = 8, the input mode is {1, 2, 3, 4, 5, 6, 7, 8}, and the sample size is chosen as *N*_*m*_ = 2 × 10^6^, which is nearly 16 times as large as the number of the collision-free events. By dealing with the sampling events, we could get the set of data *C* using equation (1). Thus, through statistical analysis, for each *U* matrix, we can get the corresponding *CV* and *S* of data *C* using the equation (2) for each sampling procedure. Figure 2 shows the outcomes for each sampling procedure, where the *x*-coordinate represents the *CV* and the *y*-coordinate indicates the *S*. In Fig. 2, each point is obtained by the statistical analysis for the sampling procedure with different *U* matrix, and the points of different colors indicate the results of different types of sampling. Similar to the previous work^37^, points for the same sampling will be gathered into a cloud, and points for different types of sampling are all separated from each other, showing clearly that different samplings can be distinguished. This phenomenon provides us a strong quantitative tool for the certification of collision-free boson sampling. In addition, compared to^37^, two more typical types of sampling (uniform sampling and thermal state sampling) are added as a comparison to show the universality of our scheme.

## Performance analysis

We have shown that a reliable validation procedure for collision-free boson sampling can be achieved. However, in the practical application, there are still some factors that a reasonable validation procedure should consider. (1) Figure 2 only gives a general regularity for large amounts of *U* matrices under the condition of a large number of samples. However, in actual experiments, we usually can only sample a small amount of samples for only a few *U* matrices. Thus, we must consider how to use this method in practical experiments. (2) How sensitive is this method? For example, perfectly indistinguishable single photons are unlikely to be achieved in the experiment. In this case, it is necessary to research the phenomenon of imperfect collision-free boson sampling.

**Figure 3:**
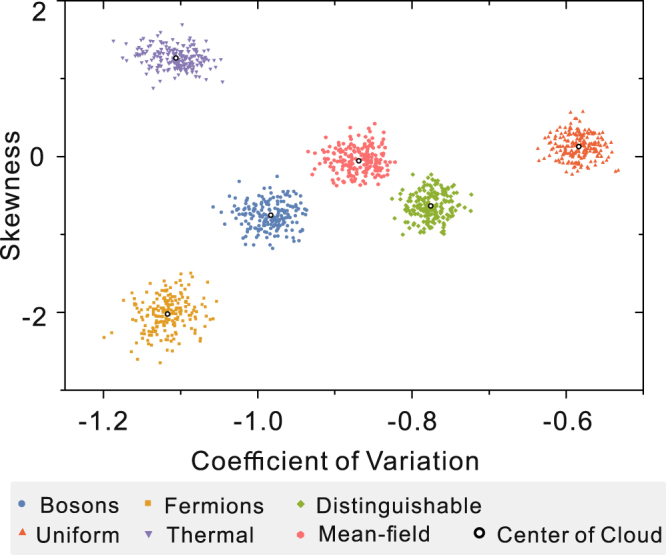
Numerical simulation results of the statistical analysis scheme with limited samples. In the simulations, *m* = 32, *n* = 5, which means total Hilbert space size of the collision-free events is *C*_32_^5^ = 201,376. For each type of sampling, we simulate 200 rounds of sampling processing, and the sample size is fixed as *N*_*m*_ = 2000 for each round. Obviously, in the case of the number of samples less than 1% of the Hilbert space dimension, different types of sampling can still be distinguished.

To apply our method to practical experiments, we should analyze the performance of our scheme for collision-free boson sampling with limited linear quantum network *U* and samples. Based on the method outlined above, without loss of generality, we simulate the experimental validation process with a randomly chosen matrix *U* and limited samples. In our simulations, we let *m* = 32, and *n* = 5, which means total Hilbert space size of the collision-free events is ${C}_{32}^{5}=201,376$. For each type of sampling, we simulate 200 rounds of sampling processing, and the sample size is fixed as *N*_*m*_ = 2000 for each round, which is a reasonable detection count in experiments. Figure 3 shows the result of the simulation, where each point is obtained from each round of sampling. Obviously, in the case of a small number of samples, different types of sampling can still be distinguished. Our simulations show that one could conclusively certify the collision-free boson sampling with number of measurements less than 1% of the Hilbert space dimension.

**Figure 4:**
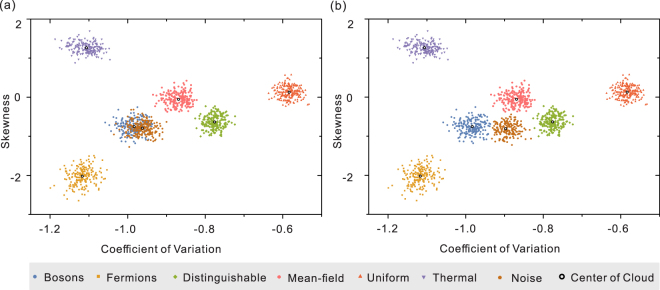
Numerical simulation results of the statistical analysis scheme with noise. In the simulations, *m* = 32, *n* = 5, and the sample size is fixed as *N*_*m*_ = 2000. (**a**) The result for imperfect boson sampling with indistinguishability *α* = 0.95. In this case, there is a large overlap between the statistical results of imperfect collision-free boson sampling and ideal collision-free boson sampling (**b**) The result for imperfect boson sampling with indistinguishability *α* = 0.8. In this case, the cloud of points obtained from imperfect collision-free boson sampling is separated from the cloud of points obtained from ideal collision-free boson sampling.

Furthermore, we also consider the effects of realistic conditions in experiments. The non-classical Hong-Ou-Mandel^43^ interference in the boson sampling multi-photon interferometry relies on a high degree of indistinguishability between the photons^26,44,45^. To quantify the sensitive of our method, here we employ our method to simulate the imperfect collision-free boson sampling with imperfect single photons as the input (see Fig. 4). In our simulation, for each of photon, we assume the indistinguishability can reach *α*, where 0 ≤ *α* ≤ 1, and *α* = 1 for perfect indistinguishable single photon. In particular, if the indistinguishability of photon is *α*, we could let the ratio of indistinguishable photons be *α*, and the ratio of distinguishable photons be 1 − *α* in numerical simulations. We note that, during the validation test, one should accept the experimental results that are included in the range of the point cloud of genuine collision-free boson sampling, and reject it if the experimental results are out of the point cloud of genuine collision-free boson sampling. As seen in Fig. 4, with small noise (*α* = 0.95, see Fig. 4a), there is a large overlap between the statistical results of imperfect collision-free boson sampling and ideal collision-free boson sampling, we will likely accept the experimental results. When the distinguishability becomes substantial (*α* = 0.8, see Fig. 4b), imperfect collision-free boson sampling will not pass the validation test, since points obtained from imperfect collision-free boson sampling are gathered as a cloud different from the ideal collision-free boson sampling.

**Figure 5:**
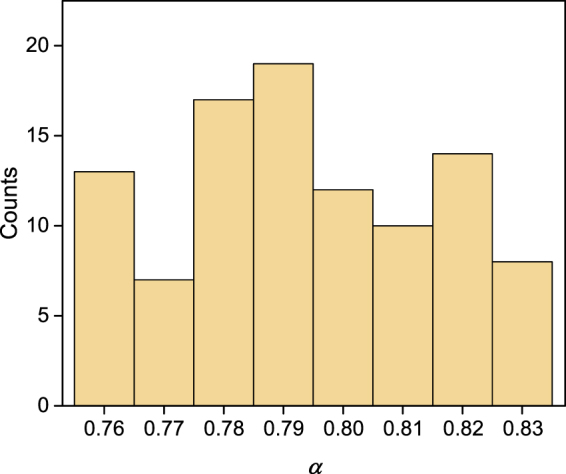
The counts of critical values of *α*. In the simulations, *m* = 32, *n* = 5. We randomly simulated 100 rounds of sampling processing for 100 *U* matrices. The mean of critical values of *α* is 0.79.

To characterize usually when the imperfect collision-free boson sampling will not pass the validation test, we need to estimate the critical value of *α*, which is determined when the cloud of points obtained from imperfect collision-free boson sampling is separated from the cloud of points obtained from ideal collision-free boson sampling. We randomly simulated 100 rounds of sampling processing for 100 *U* matrices. The counts of different critical values of *α* for these 100 rounds of simulations are shown in Fig. 5. In our simulation, the mean of *α* is 0.79, which means that the imperfect collision-free boson sampling usually will not pass the validation test when the indistinguishability *α* = 0.79. The simulation shows that our method is sensitive to strong noise and could be used as a stringent validation test.

## Conclusion

In summary, a statistical analysis scheme has been proposed to identify the nature of different types of sampling in a single framework, which opens up a new way for experimentally certifying the collision-free boson sampling with the current experimental technique. Numerical simulations are carried out to show we could conclusively certify the collision-free boson sampling with number of measurements less than 1% of the Hilbert space dimension, and our scheme is sensitive to strong noise. It would be very interesting to see whether the higher-order mode correlation function, such as three mode correlation function, could provide more information for us to study multi-particle quantum dynamics in our future work.
